# HPV self-sampling implementation strategies to engage under screened communities in cervical cancer screening: a scoping review to inform screening programs

**DOI:** 10.3389/fpubh.2024.1430968

**Published:** 2024-08-27

**Authors:** Madison M. Fullerton, Caitlin Ford, Chelsea D’Silva, Bonnie Chiang, Se-Inyenede Onobrakpor, Holly Dievert, Huiming Yang, Jason Cabaj, Noah Ivers, Sandra Davidson, Jia Hu

**Affiliations:** ^1^Cumming School of Medicine, University of Calgary, Calgary, AB, Canada; ^2^19 to Zero Inc., Calgary, AB, Canada; ^3^Alberta Cervical Cancer Screening Program, Alberta Health Services, Calgary, AB, Canada; ^4^Population and Public Health, Alberta Health Services, Calgary, AB, Canada; ^5^Community Health Sciences, University of Calgary, Calgary, AB, Canada; ^6^Women’s College Hospital Institute of Virtual Care and Systems Solutions, Toronto, ON, Canada; ^7^Department of Family and Community Medicine and Institute of Health Policy, Management and Evaluation, University of Toronto, Toronto, ON, Canada; ^8^Faculty of Nursing, University of Calgary, Calgary, AB, Canada

**Keywords:** human papillomavirus, self-sampling, cervical cancer screening, scoping review, under screened populations, community engagement

## Abstract

**Introduction:**

Human papillomavirus (HPV) testing as a method of cervical cancer screening can be performed by healthcare providers or by patients through self-sampling directly in the community, removing several barriers experienced by under screened populations. The objective of this scoping review was to determine which HPV self-sampling implementation and engagement strategies have been used to engage under screened populations (i.e., Indigenous, newcomer, and rural and remote communities) in cervical cancer screening.

**Methods:**

A scoping review was conducted searching MEDLINE, CINAHL, EMBASE, Cochrane Library, and SocINDEX from inception to August 2023. The inclusion criteria were: (1) Indigenous, newcomer, and rural and remote communities; (2) countries identified as members of the Organization for Economic Co-operation and Development; and (3) intervention included HPV self-sampling. The review was registered prior to conducting the search (https://osf.io/zfvp9).

**Results:**

A total of 26 studies out of 2,741 studies met the inclusion criteria. In-person engagement with trusted community leaders was the most widely used and accepted recruitment and engagement strategy across all three populations. Six out of seven studies with Indigenous communities distributed HPV self-sampling kits to eligible participants in person in a clinical setting for collection on site or at home. Similarly, nine of the identified studies that engaged newcomers recruited participants in person through the community, where eligible participants were either given a kit (*n* = 7) or received one in the mail (*n* = 2). Lastly, of the 10 identified studies engaging rural and remote participants in HPV self-sampling, six recruited eligible participants in person at various community locations and four used electronic medical records or registries to identify and mail kits to participants.

**Discussion:**

HPV self-sampling through in person kit distribution and mail out of HPV self-sampling kits is an effective way to increase participation rates amongst under screened populations.

## 1 Introduction

With the global call to eradicate cervical cancer, human papillomavirus (HPV) testing has been identified as one of the action pillars to increase cervical cancer screening participation (1, 2). People who develop cervical cancer are typically those that are under screened or have never been screened before (3). This includes populations such as those who do not have access to a general practitioner, ethnic minorities, new immigrants and refugees (i.e., newcomers), those of lower socioeconomic status, those who live in rural and remote areas, or identify as Indigenous (i.e., individuals who inhabited a geographical region before or during the arrival of different cultures or ethnic origins) (4–10).

Traditional cervical cancer screening uses liquid-based cytology, collected by clinicians via Papanicolaou (Pap) tests, at regular screening intervals (11). However, several barriers have been identified when receiving a Pap test such as discomfort, difficulty making an appointment, embarrassment, lack of time, and limited access to transportation (12). Therefore, HPV testing removes several of these barriers to screening as the sample can be collected by the patient themselves, either in a clinical setting or at home (i.e., HPV self-sampling). Furthermore, it has been shown that HPV testing can identify cervical pre-cancer earlier than screening with the Pap test and may lower the likelihood of developing cervical cancer (13).

Previous research has tested the feasibility and acceptability of HPV self-sampling among under screened or never screened populations, reporting that HPV self-sampling is easy to understand, complete, and participants would be willing to use this method again (14–16). Furthermore, in Canada, recent pilot projects indicate widespread acceptance of HPV testing and self-sampling among never- and under-screened (9, 13, 17) and First Nations populations (7, 18). As a result, HPV testing has gained global acceptance as the leading approach to cervical cancer screening to improve patient outcomes (19). This strategy has been adopted by countries around the world (20) including Australia (21), the United Kingdom (22), and the Netherlands (23) and, in 2024, Canadian provinces such as British Columbia introduced HPV testing as part of their provincial screening program making it available for all residents (24).

As other regions prepare to pilot or fully implement HPV testing as part of their screening programs, it is essential to understand and consider the needs of never- and under screened populations to increase cervical cancer screening participation rates. For example, the Canadian province of Alberta is preparing to pilot HPV self-sampling in 2024 amongst Indigenous, newcomer, and rural and remote communities, as these populations were identified through a series of geospatial and population specific studies conducted in Alberta to identify hot spots where cancer screening rates are the lowest (25). However, there is limited synthesized evidence on best practices to meaningfully engage these communities in HPV self-sampling. Therefore, to inform the pilot of HPV self-sampling in the Canadian province of Alberta, this scoping review aimed to answer the following questions:

1. What HPV self-sampling pilot and program implementation strategies have been used to recruit or engage Indigenous, newcomer, and rural and remote populations in cervical cancer screening?
2. What are the key considerations (i.e., barriers, facilitators, etc.) for implementing HPV self-sampling among Indigenous, newcomer, and rural and remote populations?

## 2 Methods

A scoping review was conducted following the methodological framework by the Joanna Briggs Institute (26). A comprehensive published protocol can be accessed^1^ and we reported our process according to the PRISMA Extension for Scoping Reviews (27) (Supplementary Material Table 1).

### 2.1 Search strategy

After reviewing the search terms used in previous reviews, as well as the key words from relevant peer-reviewed articles on HPV self-sampling, key search terms were piloted in three databases (MEDLINE, CINAHL, Embase) to identify seed articles relevant to the research question. The seed articles were then analyzed for index terms and text terms to ensure comprehensiveness and used to construct a final list of keywords and subject headings for the formal scoping review. A search was conducted in August 2023 from inception of the following five electronic databases: MEDLINE, CINAHL, EMBASE, Cochrane Library, and SocINDEX, to identify articles that meet the inclusion criteria (Supplementary Material Table 2). The researchers also scanned the reference lists of eligible studies, and those of relevant review articles to yield relevant articles.

### 2.2 Inclusion and exclusion criteria

Once the searches were complete, the datasets were uploaded on EndNote 20 (Berkeley, California, United States), and two researchers screened a random 10% sample of the articles in tandem for eligibility criteria. Any conflicts were resolved through discussion to reach consensus. Upon reaching inter-user agreement and consistency, the researchers then screened titles and abstract in duplicate. Conflicts were resolved through discussion, and when required, a third researcher was engaged to resolve any remaining disagreements. After screening the titles and abstracts, the researchers conducted a full text review of the articles, including hand searching the reference lists to identify additional relevant articles. Study eligibility was determined by the following criteria, guided by the PICO (Patient, Intervention, Comparison, Outcome) Framework (28):

∙ **Population:** Studies were included if their population included adults (18+), were conducted in one of the 38 countries identified as members of the Organization for Economic Co-operation and Development (OECD) (29), and from one of the following populations:

◦ Indigenous: Individuals who inhabited a geographical region before or during the arrival of different cultures or ethnic origins (10).

◦ Newcomer: ‘Newcomer’ is a heterogeneous term used to define individuals who are recent immigrants or refugees to a country (30).

◦ Rural and remote: Individuals living outside of the urban commuting zone and isolated from neighboring communities, respectively (31).

∙ **Intervention:** Studies that tested HPV self-sampling or physician-supported self-sampling via opt-in (i.e., eligible individuals are mailed a letter prompting them to register for HPV self-sampling) and opt-out (i.e., eligible individuals are directly sent an HPV self-sampling kit) for at-home testing, provider clinics, community centers, gatherings, or Community Health Ambassadors (CHA) were included. Studies that tested physician-collected samples or non-HPV cervical screening (i.e., Pap tests, etc.) were excluded.

∙ **Comparator:** Any comparator was considered appropriate for inclusion in this review (i.e., opt in vs. opt out, physician HPV testing, Pap tests, no testing, etc.).

∙ **Outcome:** Study outcomes were broad and could include testing uptake, program acceptance, participation and testing rates, identification of barriers, facilitators, attitudes, and behaviors toward self-sampling. Studies that measured cost effectiveness or non-patient-centered outcomes were excluded (i.e., health system impacts).

∙ **Study Design:** The researchers included the following study designs: Experimental and quasi-experimental study designs (i.e., randomized controlled trials, non-randomized controlled trials); observational studies (i.e., prospective and retrospective cohort studies); case–control studies and analytical cross-sectional studies; descriptive observational study designs (i.e., case series, individual case reports and descriptive cross-sectional studies); qualitative studies; editorials; gray literature including conference abstracts, reports from health authorities and healthcare organizations in Canada and other global leaders. Secondary studies—i.e., systematic reviews, meta-analyses, scoping reviews and literature reviews—were excluded.

### 2.3 Data extraction

Two researchers conducted a pilot data extraction on 10% of the articles to reach agreement and consistency. Once these parameters were agreed upon, the researchers divided the remaining articles in half, and independently extracted the following data: Title; Authorship; Year of publication; Country where the study was conducted in; Population and sample size; Research methods; Study aim/research questions; Description of intervention; Outcomes (primary and secondary); Key findings relevant to the research questions (Supplementary Material Table 3).

## 3 Results

**Figure 1:**
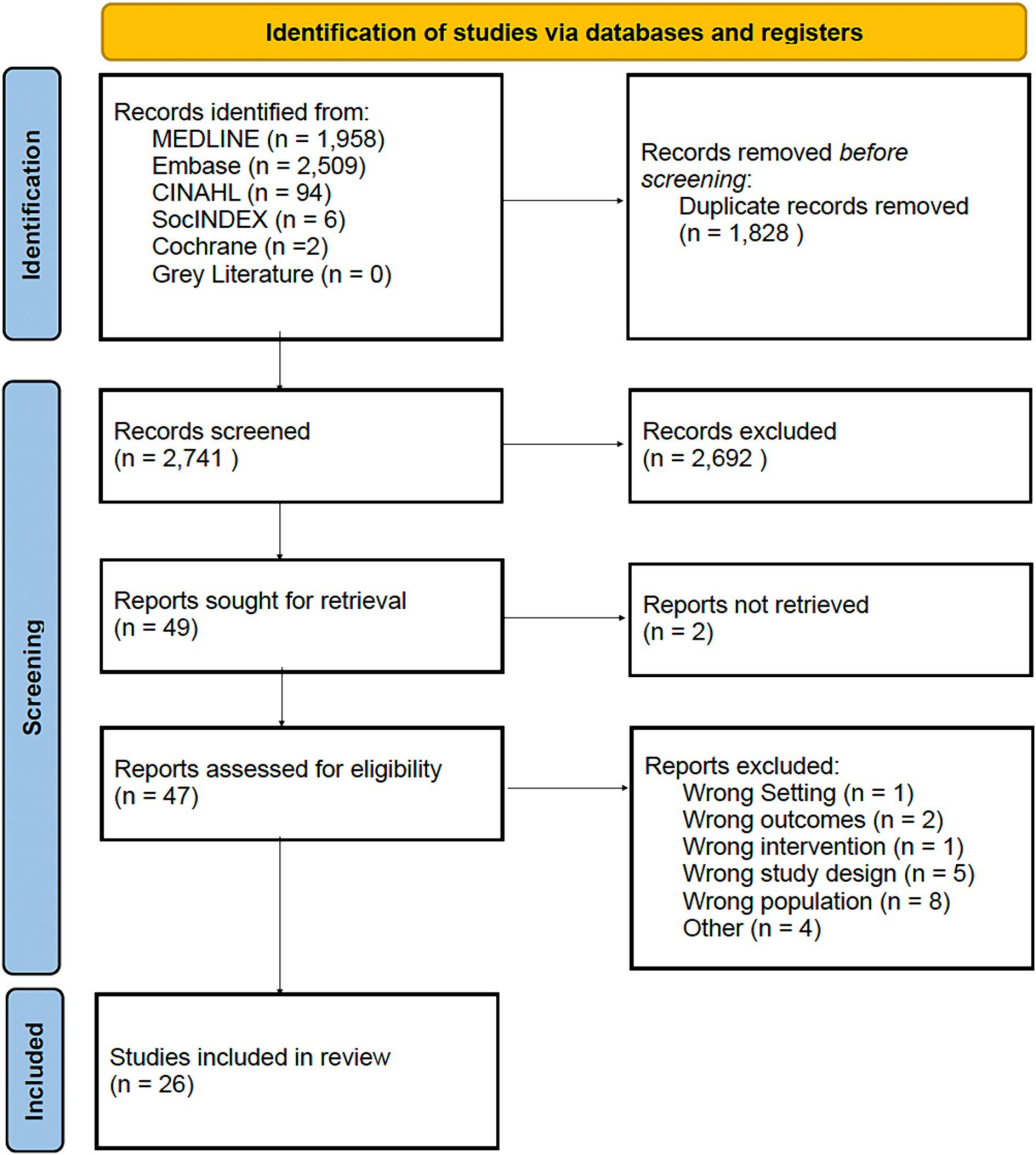
PRISMA flow diagram for systematic reviews.

A total of 26 studies met the inclusion criteria. Seven studies specifically addressed Indigenous engagement while nine and 10 addressed newcomer and rural/remote communities, respectively (Figure 1).

### 3.1 Indigenous population HPV self-sampling studies

#### 3.1.1 Population characteristics

Seven studies recruited Indigenous populations to participate in HPV self-sampling programs (*n* = 49–3,553 participants/study; Table 1) (7, 32–37). These studies were conducted in Australia (*n* = 1), Canada (*n* = 2) and New Zealand (*n* = 4). Eligible people came from Māori (*n* = 4), First Nations (*n* = 2), Métis (*n* = 1), and Indigenous Australian (*n* = 1) communities. Participants’ ages varied across studies but ranged from 25 to 69 years old.

**Table 1:** Data extraction: Indigenous population HPV self-sampling included studies.

Authors	Year	Country	Study design	Sample size	Summary of engagement	Summary of intervention	Summary of results
Dutton, et al. (32)	2020	Australia	Pilot study	215	Female indigenous community engagement workers recruited eligible Aboriginal and Torres Strait Islander participants through community events and home visits.	HPV self-sampling devices distributed at the point of recruitment. Individuals completed the self-sample with the support of the CHAs, who subsequently sent the collections to a laboratory.	The intervention was effective in reaching never- and under-screened Indigenous individuals for cervical cancer screening. The participants were highly satisfied with the self-sampling and recruitment process.
Zehbe et al. (33)	2016	Canada	RCT	834	Community health research assistants recruited First Nations participants through home visits, educational sessions, social media posts, posters, flyers, health care offices, community events and meetings.	~50% of the sample (n = 404) were assigned to the self-sampling arm and the remaining (n = 434) were assigned to a Pap test arm. The research assistants provided participants in the self-sampling arm with kits and instructions and made Pap test appointments for participants in the control arm.	Less than 25% of participants in each arm participated in screening. However, results indicated that participants preferred self-sampling over traditional pap tests.
Zehbe et al. (7)	2011	Canada	Pilot study	49	Pilot information was shared via community meetings and workshops; and flyers posted around the community and sent to all households. Eligible First Nations and Métis participants were instructed to contact the local Indigenous family health team staff if they wished to participate.	A nurse from the community family health team provided instructions for self-sampling and the participants collected the self-sample on-site. The nurse collected the sample and sent the swab to the research team.	This intervention was highly accepted by participating Indigenous individuals. Most reported that they would participate in self-sampling in the future (87.2%), and that they preferred this method to HCP sampling (67%).
Bromhead et al. (34)	2021	New Zealand	Feasibility study	84	An indigenous nurse identified and invited eligible Māori participants to participate in self-sampling at a local clinic through phone calls, text messages, letters. Participants were also recruited at focus groups. If a woman could not be reached on a first attempt, a total of five contact attempts were made.	At the clinic, the nurse provided a culturally safe consultation on the self-sampling procedure and distributed test kits. Participants had the option to collect the sample at the clinic or bring it home and return to the clinic within 7 days for analysis.	84 of the 366 contacted participants agreed to participate. All individuals who participated felt that self-sampling was easier than traditional screening and most felt that it was less embarrassing and more convenient than screening by a HCP.
Brewer et al. (35)	2021	New Zealand	RCT	3,553	Participating clinics sent the research team a list of Māori and Pasifika women who were eligible for screening from their databases. The research team randomly assigned the participants into three arms, and invited them to either collect a self-sample at their home, at a local clinic, or receive a regular cytology screening test.	Participants were assigned into three arms: Clinic (*n* = 1,574), Home (*n* = 1,467) and Control (*n* = 512). The clinic arm were invited to visit their local medical center and collect a self-sample; the home arm was mailed a kit and instructed to collect a self-sample at home; and the control arm was invited to a clinic to receive a standard cytology sample from a HCP.	Participants who received the self-sampling kit at home were significantly more likely to participate than those who were in the usual care arm. Participants who were invited to collect a self-sample at a local clinic were less likely to participate than those who received the kit in the mail.
MacDonald et al. (37)	2021	New Zealand	RCT	1730	Eligible Māori participants were identified through a patient database in each of the participating clinics. Clinical staff and local Indigenous CHAs recruited Māori individuals to participate using standard cancer screening invitation methods—i.e., calls, emails, and letters.	A subset of the sample was assigned to the control group (*n* = 806, including *n* = 431 Maori) and the remaining were assigned to the intervention/ self-sampling group (*n* = 733, including *n* = 500 Maori). The control group was invited to attend their local clinic for a standard HCP administered cytology screening test, and the intervention group was invited to collect a self-sample at the clinic, at home, or at a community center. The mode of self-sampling was arranged by the participating clinics.	Most Indigenous participants who accepted a self-sample took their own swab (90.6%), and the remaining were taken by a HCP (8.3%), or the collection method was unknown (1.1%). The majority of these participants preferred to take the self-sample on-site at the clinic (73.6%), however a large proportion chose to collect at home (22.4%).
Brewer et al. (36)	2019	New Zealand	Pilot study	56	Those eligible to participate were identified by a local health center, and community nurses recruited Māori and Pasifika participants to attend a clinic visit through mail, community outreach, home visits, at a community health promotion meeting, and at cancer screening clinics.	At the clinic, individuals were given a package with information on HPV, three self-sampling devices and written instructions for self-collection. The participants performed a self-sample with at least one of their preferred devices at the clinic and returned the test to the community nurse.	After collection, most participants (65.9%) said that self-sampling would be their preferred method of screening in the future. They found that most participants would prefer to receive the self-sampling kit at the clinic (58%) and the remaining through the mail (42%).

#### 3.1.2 Engagement and recruitment strategies

Several studies (*n* = 4) identified eligible participants through health registries or/databases, and clinical patient records (34–37). Once identified, these participants were recruited to participate in HPV self-sampling in a variety of ways. Four studies called, texted, or sent letters to eligible individuals inviting them to participate (34–37). Two of these studies also used door-to-door recruitment strategies to recruit participants (36, 37), with one study using trusted indigenous community healthcare workers to invite individuals to participate (37). In these studies, interested participants could be enrolled immediately by members of the research team if they expressed interest (36, 37).

The remaining studies (*n* = 3) identified eligible participants through a community search strategy (7, 32, 33). Two studies used community events to recruit eligible participants in real-time (32, 33). However, in combination with community event recruitment, these two studies also recruited through home visits (32, 33). One study recruited participants by distributing flyers at community events such as parenting workshops; and through newsletters delivered directly to households in the community (7). Interested participants were asked to contact relevant CHAs to enroll in the study (7).

#### 3.1.3 HPV self-sampling interventions

One study included a mailed component for HPV self-sampling kits as part of their study, where one group of participants were mailed kits to participants in an opt-out model and were instructed to self-sample and return their kits to the research team by mail (35).

All six remaining studies distributed HPV self-sampling kits to participants in person (7, 32–34, 36, 37). In total, five studies provided participants with a self-sampling kit in a clinical setting (7, 33–37). Four studies requested that participants collect the sample at the clinic and return the kit to the healthcare provider (HCP) immediately following collection (7, 33, 35, 36); and two studies gave participants the option to collect either at the clinic, or at home and return their sample to researchers later (34, 37).

Only one study provided self-sampling kits to participants at home or in community settings for immediate collection (32). These participants were given a self-sampling kit by female community engagement workers appointed by the Local Aboriginal Land Council, and were instructed to self-collect in their home, or at the community setting where they were recruited (32).

Two studies offered participants in their intervention groups the choice of a Pap test instead of an HPV self-sampling test (33, 37), however the remaining studies provided self-sampling as the only option to the intervention groups (7, 34–36).

#### 3.1.4 Participation rates

In the two studies that gave participants a choice between HPV self-sampling kits and a Pap test, self-sampling was chosen more often (33, 37). In one study, significantly more Māori participants chose self-sampling (73.6%) over Pap testing (13.9%; *p* < 0.001); and across this study population, individuals who were offered a self-sampling kit were 2.8 times more likely to participate in screening than those who were not offered (*p* < 0.001) (37). Similarly, another study found that uptake for self-sampling was slightly higher (20.6%) than Pap testing (16.0%), when both were offered to Indigenous participants, though results were not significant (*p* = 0.694, 33).

The one study that used mailed-out kits found that participation rates in Māori individuals that were mailed a kit to their homes were significantly higher than those offered regular care (35). They determined that Māori individuals who are mailed a self-sampling kit were 10 times more likely to participate in screening than those who were assigned regular care (i.e., Pap test; Odds Ratio = 9.7; 95%CI 3.0-31.5) (35).

Four studies explored outcomes that were not directly related to participation rates (32–34, 36). These studies were not designed to report on participation rates, because all individuals enrolled in the study completed a self-sample (32–34, 36). Instead these studies assessed factors like acceptability (32, 34, 36), positivity rates (33), and feasibility (34), which were not captured in the scope of this review.

#### 3.1.5 Barriers and facilitators to self-sampling

HPV self-sampling was generally accepted as a screening tool for cervical cancer. In several studies, participants reported that self-sampling was an easy and convenient way to screen for cervical cancer (7, 32, 34, 36). Three studies found that participants would consider mail-out options to be acceptable as a means for reducing any barriers to accessing a clinic (32, 35, 36). In addition, four studies identified the importance of having an Indigenous healthcare worker or community ambassador to help build trust among study participants (7, 33, 34, 37).

### 3.2 Newcomer population HPV self-sampling studies

#### 3.2.1 Population characteristics

Nine studies were identified where the target population were newcomers who were eligible for cervical cancer screening (*n* = 64–1,213 participants/ study; Table 2) (14–16, 38–43). Participants were living in the United States (*n* = 8) or Canada (*n* = 1). Study participants identified as Haitian, Hispanic, Chinese, Vietnamese, Korean, Middle Eastern, North African, Black, and Somali.

**Table 2:** Summary of newcomer population HPV self-sampling studies.

Authors	Year	Country	Study design	Sample size	Summary of engagement	Summary of intervention	Summary of results
Devotta et al. (38)	2023	Canada	Community-based mixed methods	108	Recruitment was conducted by CHAs (i.e., those who were of West/South Asian, Middle Eastern or North African descent, and related to local community organizations). Participants mainly consisted of immigrants who had moved to Canada within the past 20 years (i.e., Canadian citizen by naturalization, landed immigrant/permanent resident, refugee/refugee applicant).The CHAs recruited through local groups, places of worship, community/ cultural events, flyers, and in-person presentations.	Participants who elected to participate in self-sampling were given a test kit in a postage paid return package—distributed both in person, and through the mail. The kit contained instructions for self-sampling, and the participants were instructed to return the kit via mail. Those who chose not to participate in self-sampling were encouraged to receive a pap test.	61 participants completed the self-sample and mailed it back to the research team. The participants reported that the self-sampling kit begins to remove privacy and comfort barriers, however they expressed that clearer instruction, and more support was needed for collecting a sample at home.
Ma et al. (39)	2022	USA	Pilot Study	156	Participants who self-identified as Chinese, Vietnamese, or Korean were recruited through flyers distributed at faith-based and community organizations. The flyers were available in three different languages and instructed participants where/when to attend if they wanted to participate in self-sampling.	Bilingual CHAs provided educational workshops at the faith and community-based organizations and provided participants with a self-sampling kit and instructions on how to use them. Participants then completed the self-sample in a private bathroom on-site.	All participants who received the educational session chose to participate in self-sampling. Most participants (74%) felt confident in their ability to collect a self-sample again after completing the test.
Carrasquillo et al. (40)	2018	USA	RCT	601	Local CHAs recruited participants—majority of whom were Hispanic and Haitian immigrants—at community venues (i.e., stores, community events, churches). Participants who expressed interest were subsequently contacted by the research team to schedule an intake visit at either the participant’s home, a community health center, or another mutually agreed upon community venue. After recruitment, participants were assigned to three groups: outreach, navigation, or self-swab.	Participants in the outreach group were given a culturally tailored brochure on cervical cancer screening, and how to receive a pap test, in their preferred language (English, Spanish, or Haitian Creole). Participants in the navigation group received the brochure, along with a 1:1 education session with a CHAs to reinforce the educational materials, and assistance in booking a Pap test. The self-swab group received the same educational interventions as the navigation group but were instead offered an HPV self-sampling kit and invited to perform the test at the time of the education visit, or, had the option of having the health worker help them book a Pap test.	The self-sampling group had an increased rate of screening than did the other groups, and in total, 265 participants completed the self-sampling test. The participants in this group exhibited a higher degree of cervical cancer knowledge than other groups, due to their active role in the screening process.
Kobetz et al. (41)	2018	USA	RCT	600	CHAs recruited participants who identified as Haitian and Hispanic immigrants as well as African Americans at community churches, flea markets, and events. Interested participants then scheduled a meeting with a health educator at their home or a different agreed-upon where participants were randomly assigned to the in-person self-sampling, or mail-out self-sampling arms.	The in-person group was given an educational session by the CHAs on cervical cancer screening and instructions on self-sampling. The participants were then given the self-sampling kit and were given an option to collect on-site while the health worker waited, or to self-sample at home and return the kit using pre-paid postage. The mail-out group was sent a self-sampling kit including instructional materials, and a pre-paid postage to return. One week after the kit was mailed, the CHAs phoned the participants and provided a brief educational session.	Uptake of HPV self-sampling was high in both groups; however, it was significantly higher for the in-person group (81%, *n* = 243) compared to the mail-out group (71.6%), *n* = reaching 81.0% (*n* = 243) among the in-person participants and 71.6% (*n* = 214) among mail-out.
Ilangovan et al. (14)	2016	USA	Pilot study	180	Participants were recruited from an area with a high density of Haitian immigrants by CHAs—females of Hispanic and Haitian origin—in the waiting rooms of two local health clinics.	Participants were given a culturally sensitive educational session on cervical cancer and self-sampling by the CHAs. The participants were given a choice to perform a self-sample on site or discuss the Pap smear option with a HCP.	Most participants who were offered self-sampling accepted (67%). Almost all participants who self-sampled (99%) considered it to be an acceptable method for screening.
Sewali et al. (42)	2015	USA	RCT	64	A Somali health team recruited Somali immigrant participants by word of mouth, and flyer distribution. Interested individuals then participated in a 60-min information session at a community center or at home, led by one of the health team staff, where participants were assigned into the self-sampling or Pap test arms.	The self-sampling arm was provided with a test kit along with instructions for completing the self-swab at home. The participants were told to return the test kit to the clinic within 3-months. The Pap test arm was asked to follow up with HCP to schedule a Pap test within 3 months.	Participants who received the self-sampling kit were more likely (65.6%) than the pap test group (19.4%) to follow through with cervical cancer screening. Most participants reported that the self-sampling instructions were easy to follow, and if given a choice they would prefer to self-collect than to receive a Pap test.
Montealegre et al. (15)	2015	USA	Pilot study	100	Mexican immigrants were recruited to participate through announcements made on the intercom in the waiting room of the consulate. Interested participants could approach the study kiosk at the consulate to enroll. Additional participants were referred to this pilot study by staff at a Latino health promotion organization.	Once enrolled, participants were provided a self-sampling test kit at the consulate and were instructed to collect a self-swab on-site in the private restroom. The swab collections were then returned to study staff for analysis.	Most participants felt that the instructions were easy to follow (98%) and that the test kit was easy to use (83%).
Barbee et al. (16)	2010	USA	Community- based participatory research	246	CHAs recruited Hatian immigrants at local venues including flea markets, health centers, laundromats. If participants were eligible and interested in participating, the workers scheduled a follow-up meeting for them to collect the self-sample.	The CHAs provided an educational one-on-one session at the participants’ homes where they provided information on HPV and instructions on self-sampling. Participants then collected the samples in private and gave the collection to the health worker.	The participants were highly satisfied with the self-sampling process. Most felt comfortable with the device (97.6%), would recommend self-sampling to their friends/family (98.4%), and felt that the test kit was easy to use (95.1%).
De Alba et al. (43)	2008	USA	Feasibility study	1,213	Health workers from a Latino community health center recruited Hispanic participants through flyers, newspaper ads, invitations at health fairs, public spaces, home visits, and cancer screening presentations at schools, churches, and community-based organizations.	Eligible participants were provided a self-sampling kit with Spanish instructions. Participants were instructed to perform the self-sample at home, in the bathroom at the point of recruitment, or in a preferred location. Once swabs were collected, they were returned to the health worker.	Most participants were satisfied with the self-sampling (64%). They felt that the self-collection procedure was convenient and easy to complete.

#### 3.2.2 Engagement and recruitment strategies

Of the studies that looked at HPV self-sampling amongst newcomer communities, all nine used in-person engagement to identify and recruit eligible participants. Eight studies used individuals who were familiar or identified with the communities they served—classified as “community champions” (38), “community health workers” (14, 16, 39, 40, 42, 43), or “community health educators” (41)—to recruit eligible participants. In comparison, one study recruited participants through staff and public announcements in the waiting area of a Consulate (15).

Through CHAs, several approaches were used to engage newcomers including direct (i.e., presentations, door-to-door, canvassing, etc.), or passive (i.e., flyers, posters, advertisements in the newspaper, etc.) recruitment at various locations in the community such as neighborhood associations, tea parties, parent groups (38); community events like health fairs (38–43); places of worship (38–41); stores or other public spaces (i.e., laundromats) (16, 40, 42, 43); public health clinics (14, 16); and flea markets (16, 41).

#### 3.2.3 HPV self-sampling interventions

After identifying eligible participants, CHAs then provided education on HPV self-sampling and cervical cancer at locations including: the homes of participants, community organizations, or other mutually agreed upon locations. At these locations, participants were given the option to either immediately self-collect with the support of a CHA (16, 39, 40), self-collect later (i.e., at home) (38, 42), or both (40, 43).

In addition to distributing the kits in-person, two studies also sent HPV self-sampling kits directly to the homes of eligible participants who were identified through various engagement strategies and opted-in to participate (38, 41). Furthermore, in the study by Kobetz et al. (2018), participants who received their HPV self-sampling kit in the mail also received HPV education over the phone by a CHA. Those that self-collected at home were instructed to either return the HPV self-sampling kits in a prepaid envelope via mail (38, 41) or return it to the CHA (42, 43), for laboratory processing.

Two studies recruited participants at either a Consulate (15) or public health clinics (14), and immediately provided eligible participants with the HPV self-sampling kit for collection on-site. When participants were recruited through the public health clinics, the CHA identified as either Latina or Haitian and were instructed to only recruit Latina or Haitian patients (14).

Education and materials provided to participants about HPV self-sampling were provided in multiple languages and the CHAs were able to verbally translate and communicate with participants (15, 16, 38–42).

#### 3.2.4 Participation rates

Overall, in-person community education and recruitment through CHAs were highly effective at engaging eligible participants in HPV self-sampling. A study by Kobetz et al. found that when newcomers were engaged and given an HPV self-sampling kit in-person, participation rates were higher (81.0%) than for those that were sent a kit in the mail after being engaged in person (71.6%; *p* < 0.01, 41). Carrasquillo et al. reported 64.0% participation when newcomers were recruited at locations in the community such as stores and places of worship (40). One study by Ilangovan et al. reported 67.0% participation when CHAs were used to recruit participants of the same ethnicity at a public health clinic (14). Carrasquillo et al. also reported participation rates of 76.0% amongst a group of participants who were not allocated to the HPV self-sampling arm of the study but were given the option to participate once the study was over (40). Studies by De Alba et al. and Ma et al. reported that 1,213 and 156 newcomers participated in HPV self-sampling, respectively, through in-person recruitment at community events, places of worship, etc., but did not report the number of people who were invited to participate (39, 43). Therefore, we are unable to assess the participation rate.

When comparing HPV self-sampling participation rates to Pap testing, Sewali et al. found that newcomers were more likely to participate in cervical cancer screening via self-sampling (65.6%) compared to Pap testing (19.4%) within a clinical setting (*p* = 0.0002) (42). Similarly, Devotta et al. found that 61 participants mailed back their sample and only 23.6% of the participants who chose not to participate in self-sampling went for a Pap test (38). Lastly, studies by Montealegre et al. and Barbee et al. were not designed to report the participation rates (15, 16).

#### 3.2.5 Barriers and facilitators to self-sampling

Overall, the use of CHAs made participants more confident to perform self-collection and likely resulted in high participation rates (14, 16, 38, 41). Participants also expressed that through the education sessions they gained a greater knowledge of cervical cancer and the importance of screening (40), and appreciated the convenience and privacy of the test compared to the Pap test (15).

However, a few barriers were noted which included worry that the test would be uncomfortable, concerns of how their partner (i.e., spouse) would react (38); and fear of sampling incorrectly (15, 16, 38). The study by Kobetz et al. also found that participants were uncomfortable mailing their samples at government-run post offices due concerns around immigration status (41).

### 3.3 Rural and remote population HPV self-sampling studies

#### 3.3.1 Population characteristics

In total, 10 studies were identified where the target population were individuals eligible for cervical cancer screening residing in rural and/or remote areas in Canada (*n* = 3), Denmark (*n* = 1), Greece (*n* = 2), Japan (*n* = 1), and the United States (*n* = 3) (*n* = 31–13,111 participants per study; Table 3) (9, 17, 44–51). Researchers defined the study population’s eligibility as those living in rural or semi-rural area codes and regions, those who are geographically isolated, or those living in remote areas.

**Table 3:** Summary of rural and remote population HPV self-sampling studies.

Authors	Year	Country	Study design	Sample size	Summary of engagement	Summary of intervention	Summary of results
Jalili et al. (17)	2019	Canada	Cohort study	1,052	Eligible participants were identified on an EMR and were randomly assigned to the self-sampling, or control groups. Those eligible to participate were contacted by mail.	Participants in the self-sampling group received an at-home test kit by mail, including instructions to collect and send the kit back. Participants in the control group received no communication from the research team during the study period.	More than 86% of individuals who were contacted did not respond to the invitation to participate. After a six-month study period, 9.6% of participants in the self-sampling group returned their self-test and 1.1% of participants completed a Pap test.
Racey et al. (9)	2016	Canada	RCT	818	Eligible participants were identified from a clinic’s EMRs. Eligible participants received mail inviting them to participate and were randomly allocated to one of three groups: self-sampling, reminder letter, or standard of care.	Those in the self-sampling arm were sent a letter from the clinic outlining the study, followed by an at-home self-sample kit 2 weeks later to be collected and sent back. Participants in the reminder letter arm received an invitation by mail instructing them to call the clinic to book a Pap test. Participants in the standard of care arm did not receive any contact from the clinic during the study period.	Both the self-sampling and the reminder letter groups were more likely than the control group to follow through with testing. However, the self-sampling group experienced the greater uptake for screening (3.7 times more likely than control), compared to the reminder letter (1.8 times more likely than control). Most participants (90%) reported that they would prefer to self-sample in the future.
Duke et al. (44)	2015	Canada	Cohort study	6,057	Three communities participated in this study and community A, B and C were assigned self-sampling + education, Pap-test + education, or control, respectively. A promotional campaign was launched in community A and B, and participants were invited to pick up self-sampling kits at locations around the community (community A) or schedule a Pap test (community B).	In community A, self-sampling kits were made available at hospitals, pharmacies, hair salons and women’s exercise centers, and a research nurse was also available to drop off kits at a woman’s home or work. Participants were instructed to drop the test kit off at a set location, or the research nurse would pick up the test kit from the participants. Participants in community B received promotional campaigns about Pap tests and cervical cancer and were encouraged to undergo a Pap test. Participants in community C received no additional promotion beyond their current standard of care.	Uptake was low for self-sampling, and only 9.5% of eligible individuals participated. Overall, however, acceptability among those who self-sampled was high, and 67.9% of participants reported that they were very satisfied with the process.
Tranberg et al. (45)	2018	Denmark	RCT	9,791	Health registry data was used to identify eligible individuals and these participants were randomly assigned to a self-sampling opt-out, self-sampling opt-in or control group.	Participants in the opt-out group were mailed a self-sampling kit along with instructions for sampling and returning the sample. Participants in the opt-in group were mailed an invitation to opt-in to self-sampling and receive a test kit by mail (i.e., opt in by email, text, phone, or website) Participants in the control group were sent a standard letter reminding them to attend regular cytology screening.	The opt-out option yielded the greatest participation rate for screening than the other two groups. Participants in rural areas were 12.3% more likely to complete screening for the opt-out method versus control.
Agorastos et al. (46)	2019	Greece	Cross-sectional study	13,111	Using a combined model of community campaigns, public announcements and door-to-door approaches, participants were invited to collect a self-sample.	Participants were provided with a self-sampling kit and were instructed to collect the sample—either at home or in a primary care facility—and return the swab to the study team.	In total, 13,111 individuals participated in this program. Participant acceptability and satisfaction were not evaluated.
Chatzistamitou et al. (47)	2017	Greece	Pilot study	346	A public announcement invited individuals living in rural areas to visit local primary health care units at a particular date, where midwives provided information on benefits of self-sampling for cervical cancer screening and the study rationale.	People that chose to participate were given a self-sampling kit on-site and were instructed to collect the self-sample alone, or with midwife supervision.	Most participants reported that they preferred self-sampling to physician-collected screening. Furthermore, most participants reported that they did not experience any discomfort (82.4%) or difficulty (77.6%) with self-sampling.
Yamasaki et al. (48)	2019	Japan	RCT	249	People identified by the local government as non-attendees for cervical cancer screening were sent an invitation letter to participate in the study. Those interested in participating were assigned to either the self-sample or re-call groups	The self-sample group was sent a self-sample kit with instructions and prepaid return postage. The re-call group was sent an invitation to schedule a standard cytology screening at a local hospital.	Participation in screening was significantly higher for the self-sampling group (76%) compared to the recall group (12.1%).
Crosby et al. (49)	2017	USA	Exploratory Study	88	Information about the project was shared during worship services at local churches. Participants were instructed to talk to female study staff after the service if they were interested in participating.	At the churches, participants were provided with a self-sampling test kit and were given verbal instructions for collecting. The participants were instructed to complete the sample in the bathroom and return the specimen to the research assistant.	Self-sampling was highly acceptable to participants, and 78.4% reported that they would prefer to repeat a self-sampling test rather than a Pap test.
Crosby et al. (50)	2015	USA	Feasibility study	400	People were recruited to participate through informational flyers and word-of-mouth at local health departments, during community outreach events, and at non-traditional healthcare settings—including mental health and substance use clinics.	At the recruitment venue, participants were provided with a self-sampling test kit and were given verbal instructions for collecting. The participants were instructed to complete the sample in the bathroom and return the specimen to the research assistant.	Self-sampling was highly accepted by the participants, and 89% preferred this method over traditional cervical cancer screening.
Vanderpool et al. (51)	2014	USA	Exploratory Study	31	A study nurse recruited participants at a local free primary care clinic and invited them to participate in self-sampling.	Participants were provided verbal and graphic-based instructions for collecting a self-sample and were instructed to collect the swab in a private bathroom at the clinic.	All participants who were approached at the clinic accepted the self-sampling kit and followed through with screening.

#### 3.3.2 Engagement and recruitment strategies

Half of the included studies (*n* = 5) used a form of in-person community engagement to identify and recruit eligible participants for HPV self-sampling (44, 46, 47, 49, 50). Methods of community engagement varied across studies, but these strategies included education and handing out kits through midwives (46) or nurses (44); visiting the homes of people in rural and remote areas (44); recruitment during worship services at rural and remote churches (49); during community outreach events and in non-traditional healthcare settings such as mental health clinics and substance treatment centers (50); at community settings like hair salons and women’s exercise centers, hospitals and pharmacies (50); and public messaging or campaigns to notify eligible participants of where they could go to access an HPV self-sampling kit (i.e., local primary healthcare units) were also used to recruit participants (46, 47).

Four studies also identified eligible people to participate in HPV self-sampling through electronic medical records (EMRs) or registry data (9, 17, 45, 48). These studies all used mail-out options (opt-in or opt-out) to invite individuals to participate. The least used engagement strategy among studies of those living in rural or remote areas was recruitment through a primary health care facility (*n* = 1, 51). Participants in this study were approached by a nurse during their visit to a free health care facility and were asked to self-administer an HPV test.

#### 3.3.3 HPV self-sampling interventions

The same studies that recruited participants through EMRs or registries (9, 17, 45, 48) provided HPV self-sampling kits by mail and instructed them to return these kits through pre-paid postage (*n* = 4). However, these studies varied in their approaches–where some sent kits to all eligible individuals in an opt-out method without prior communication (9, 17, 45), and others sent invitations for participants to opt-in to this sampling method by providing consent through the mail or registering for a HPV self-sampling kit via email, text message, phone, or through a website (45, 48).

In contrast, many studies (*n* = 6) provided participants with the self-sampling kits at the point of recruitment and/or care (44, 46, 47, 49–51). In five studies, the participants were provided a self-sample kit by the person recruiting them and were instructed to self-collect at home (46), in public or private washrooms (49, 50), or at a clinic (47, 51). In one study, participants were invited to attend a clinic where the self-sampling kit was provided to them to self-collect under supervision of a midwife, or privately in the bathroom (47). Under these circumstances, the self-collected swabs were immediately given back to researchers for subsequent laboratory analysis. However, in contrast, one study that provided self-sample kits at the point of recruitment did so by distributing kits in public areas for participants to bring home, self-collect, and mail back to the research team for analysis (44).

#### 3.3.4 Participation rates

Mailed-out self-sample kits consistently yielded greater participation rates than control groups who received routine care (i.e., those who were prompted to participate in a Pap test). One study found that when compared with a reminder to receive a Pap test, or no reminders at all, participants who received an HPV self-sample kit in the mail were 3.7 times more likely to participate in screening (9). Likewise, another study found that in comparison to Pap test participation (1.1%), mailed out HPV self-sample kits yielded a higher participation rate (9.6%); note that this rate reflects participation in both urban and rural areas however 27/50 participants total were from rural areas (17). Furthermore, one study found that in comparison to participation rates for individuals who received standard Pap test recall letters (12.6%), individuals who received the HPV self-sample kits in the mail were more likely to participate (76%) (48).

When provided with HPV self-sampling kits at locations such as hospitals, pharmacies, hair salons, and women’s exercise centers, 20.1% of those who obtained kits from these locations mailed them back to the research team (44). One study found that in comparison to the control group, an opt-out model yielded 12.3% higher participation rates, and in addition, participation rates in the opt-out model were 6.6% higher than an opt-in model (45). Similarly, another study reported high participation rates where 97.5% of identified individuals were eligible and participated in HPV self-sampling (46). Four studies were not designed to report on participation rates. Two of these, all individuals enrolled in the study completed a self-sample (47, 51) while the other two studies, recruited participants via community outreach and other non-traditional healthcare settings (49, 50).

#### 3.3.5 Barriers and facilitators to self-sampling

Overall, participant-reported barriers to HPV self-sampling were limited across the included studies. Three studies reported that a subset of individuals in their sample populations felt pain or discomfort during the self-sampling procedure (47, 49, 50). One study also noted that some of their participants had minimal trust in the healthcare system, which could serve as a barrier for uptake (51). However, the acceptance for self-sampling was generally high among participants in the included studies (9, 17, 44–51).

## 4 Discussion

This scoping review examined several HPV self-sampling engagement and implementation strategies to increase cervical cancer screening participation among Indigenous, newcomer, and remote communities. For all three populations, in person recruitment (~70% of studies) was highly successful at identifying eligible participants; however, registries and EMRs were also used to identify eligible participants from Indigenous and rural and remote communities (~30% of studies; Tables 1–3). Regardless of the strategy used to identify participants, in-person kit distribution, mail-outs, or a combination of both methods were used among all three populations regardless of sample size. Additionally, in-person distribution resulted in generally greater uptake than mail-outs—especially when CHAs were used to engage directly with participants. Overall, participants among the identified studies were receptive to HPV self-sampling and would complete this form of screening again in the future.

### 4.1 Key considerations when engaging indigenous, newcomer, and remote communities in HPV self-sampling

#### 4.1.1 Indigenous populations

HPV self-sampling has been identified as an easy, convenient, and an acceptable way to screen for cervical cancer among Indigenous populations (Table 3) (7, 32, 34, 36). Like newcomer and rural and remote engagement, included HPV self-sampling studies that utilized tailored engagement and education carried out by trusted voices from the community had successful participation rates and cited CHAs as an important channel for delivering the program (7, 33, 34, 37). Indigenous communities face several barriers when accessing health services such as geographical isolation, low health literacy, health system biases and racism (52); thus, CHAs have been proposed as an effective medium to reduce barriers and facilitate trusted health conversations (53). The use of CHAs is considered highly important for building and maintaining trust among underserved populations, and this peer-to-peer model has been proposed to increase the uptake of other cancer screening programs—such as breast and colorectal—in Indigenous communities (53). Additionally, while few studies have explored mailed-out approaches with Indigenous communities (36), participants indicated that mailing kits directly to their homes would be an acceptable way to reach them as it reduces access barriers by eliminating the need for clinic visits, which are mandatory for current screening practices (i.e., Pap test) (34–36). Ultimately, future HPV self-sampling studies (i.e., embedded within screening programs) should consider a combination of both in person engagement through CHAs as health advocates and explore participation through mail-out approaches which may be an acceptable way of engaging Indigenous communities in HPV self-sampling.

#### 4.1.2 Newcomer populations

Unlike Indigenous and rural and remote participant identification, in-person engagement was the only method used to identify and recruit eligible newcomers to participate in HPV self-sampling in the identified studies - medical registries and EMRs were not used, which may be a function of how immigration data is stored and managed. For example, in Canada, the delivery of health services (i.e., provincial screening programs) is the responsibility of provincial governments which have their own health databases (54). In comparison, immigration data is managed nationally by the Immigration, Refugees and Citizenship Canada (IRCC); and unfortunately, not every province can link health and immigration data (54). As a result, it can be challenging to systematically identify newcomers who are eligible for cancer screening and may provide one explanation for why the identified studies in this review used direct engagement in the community to identify and recruit newcomers to participate in HPV self-sampling. Additionally, in-person engagement reduces several barriers faced by newcomers when accessing screening services such as limited access to primary care services, limited social support, and lack of knowledge on cervical cancer screening (55, 56). Therefore, it is not surprising that the implementation of CHAs to support and educate newcomers on HPV self-sampling resulted in participants feeling more confident to perform self-collection and resulted in high participation rates (14, 16, 38, 41). A recent study by Lofters et al., found that CHAs were highly successful at supporting and encouraging eligible newcomers to participate in HPV self-sampling by serving as trusted voices within their communities, conversing in first languages and having healthcare backgrounds (57). Like Indigenous engagement in cancer screening, CHAs have also been successful at engaging newcomers and other underserved populations in breast and colorectal cancer screening (58, 59). This provides important considerations when designing future HPV self-sampling engagement strategies that aim to eliminate barriers to screening such as language barriers which continue to be a main factor inhibiting newcomers from participating in cancer screening services (60). Ultimately, better integration and utilization of community and social services is crucial for supporting and increasing newcomer engagement in HPV self-sampling.

#### 4.1.3 Rural and remote populations

Acceptance of HPV self-sampling was generally high among rural and remote communities, and participation rates were even higher when eligible individuals were engaged directly in the community compared to indirect approaches (i.e., mail-outs; Table 2). Other than one study with an Indigenous population (36), indirect approaches for kit distribution (i.e., direct mailouts) were only used with rural and remote populations within the identified studies. Interestingly, in-person community engagement was the most widely used strategy to recruit and distribute self-sampling kits to eligible rural and remote participants (44, 46, 47, 49–51). Like the identified studies with Indigenous and newcomer populations, the use of CHAs enabled direct and immediate education of cervical cancer screening and ease of access to self-sampling kits by utilizing pre-existing infrastructures in the community (i.e., churches, hair salons, etc.), making it easily accessible and convenient to be screened. As mentioned, CHAs are highly effective at improving the timely completion of breast, colorectal, and cervical cancer screening among underserved communities such as individuals living in rural and remote regions by bridging the gap between community needs and access to health services (61, 62). This may explain why the use of CHAs yielded higher participation rates compared to mail-out methods with rural and remote populations, however this requires further investigation. Regardless, both in person community engagement and mail-out options reduce key barriers faced by rural and remote communities when accessing health services such as barriers associated with geographical isolation and limited transportation (63). Like future work with Indigenous communities, future HPV self-sampling projects with rural and remote populations should consider ways to utilize the trusted voices of CHAs combined with mail-out approaches as alternatives to Pap testing (64).

### 4.2 Strengths and limitations

The scoping review has several strengths. Five databases were included in the search and most notably, two reviewers independently screened the articles and confirmed inter-rater reliability. A scoping review was selected for this review as it is a useful method for examining and mapping emerging evidence by identifying knowledge gaps, types of available evidence, understanding how research on a specific topic is conducted, etc. (65). Unlike systematic reviews, for example, scoping reviews do not necessarily provide a comprehensive analysis of the strength of evidence or the effectiveness of the interventions (65), however, that was not the purpose of this review.

The scoping review has several limitations. Most notably, Indigenous, newcomer, and rural and remote communities have varying definitions globally; and although these communities make up a large majority of the underscreened populations in some OECD countries, this may not be the case across all OECD countries. Another important limitation is that while this review focused on the strategies used to engage underscreened communities in HPV self-sampling, there are several other key considerations along the screening pathway (i.e., communication of results, clinical follow-up, etc.) that are important to understand when tailoring and implementing HPV self-sampling among diverse communities. Other limitations such as the inclusion of both qualitative and quantitative studies, make it harder to draw clear comparisons or conclusions across included studies. Lastly, this review was limited to papers written in English, which may have excluded key papers that focus on populations who speak several first languages.

## 5 Conclusion

Overall, Indigenous, newcomer, and rural and remote communities are accepting of HPV self-sampling as it reduces several barriers they experience when accessing health services such as cancer screening programs. Regardless of the method used to distribute the self-sampling kits (i.e., in person versus mail-out), the use of CHAs and pre-existing community events and infrastructures (i.e., churches, hair salons, clinics) was highly successful at increasing cervical cancer screening participation rates among all three populations. Evidently, CHAs are an important consideration in the implementation of HPV self-sampling; however, it is important to highlight that CHAs are regularly tasked to educate on multiple health topics at once and rarely have time dedicated to a single health topic (66). This may have implications on the integration of CHAs delivering health services directly in the community outside of pilot programs where dedicated resources and support are allocated to CHAs, emphasizing the importance of building capacity for health systems to support their integration (61). Additionally, this review highlights the importance of meaningful community engagement through tailored cervical cancer screening education that is culturally relevant and available in first languages. Future HPV self-sampling projects should take the time to understand the unique needs and barriers experienced by the populations they wish to engage and tailor their approaches accordingly. For example, distributing self-sampling kits in person, through mail-outs, or a combination of both approaches may all be appropriate strategies depending on the population and their previous experiences with screening services. Overall, participant engagement and recruitment is one of several important steps in the cancer screening pathway for achieving equitable participation and outcomes for underscreened populations through HPV self-sampling.

## Data Availability

The original contributions presented in the study are included in the article/Supplementary material, further inquiries can be directed to the corresponding author.

## Footnotes

^1^ https://osf.io/zfvp9
