# Supplementary material for: HPV self-sampling implementation strategies to engage under screened communities in cervical cancer screening: a scoping review to inform screening programs
Source: Front Public Health. 2024 Aug 27;12:1430968. doi: 10.3389/fpubh.2024.1430968 (PMC11384595; doi:10.3389/fpubh.2024.1430968)
Supplement: Supplementary file 2 [file Table_2.docx]

| **Database** | **Key Concept** | **Search** | **Terms** |
| --- | --- | --- | --- |
| MEDLINE | HPV & Cervical cancer | 1 | Exp Human papillomaviruses/ |
|  |  | 2 | Exp Uterine cervical neoplasms/ |
|  |  | 3 | Papillomavirus Infections/ |
|  |  | 4 | HPV or human papillomavirus or papillomavirus infections or cervical cancer.tw,kf. |
|  |  | 5 | 1 OR 2 OR 3 OR 4 |
|  | Self testing | 6 | Exp self-testing/ |
|  |  | 7 | ((self or at-home or home or at home or self-administered or self-collect*) adj5 (test* or screen* or sampl* or swab* or collect*)).tw,kf. |
|  |  | 8 | 6 OR 7 |
|  | Combining concepts | 9 | 5 AND 8 |
| CINAHL | HPV & Cervical cancer | 1 | (MH "Human Papillomavirus Viruses") |
|  |  | 2 | “HPV” or “Human Papillomavirus” |
|  |  | 3 | (MH "Cervical Intraepithelial Neoplasia+") OR (MH "Uterine Neoplasms+") OR (MH "Cervix Neoplasms+") |
|  |  | 4 | S1 OR S2 OR S3 |
|  | Self testing | 5 | (MH "Self-Testing") |
|  |  | 6 | (MH "Home Diagnostic Tests") OR "((self or at-home or home or at home or self-administered or self-collect*) adj5 (test* or screen* or sampl* or swab* or collect*))" OR (MH "Self Administration+") |
|  |  | 7 | S5 OR S6 |
|  | Combining concepts | 8 | 4 AND 7 |
| EMBASE | HPV & Cervical cancer | 1 | HPV.mp. or exp Wart virus/ |
|  |  | 2 | cervical cancer.mp. or exp uterine cervix cancer/ |
|  |  | 3 | “Human papillomavirus".mp. |
|  |  | 4 | 1 OR 2 OR 3 |
|  | Self testing | 5 | exp self-testing/ |
|  |  | 6 | ((self or at-home or home or at home or self-administered or self-collect*) adj5 (test* or screen* or sampl* or swab* or collect*)).tw,kf. |
|  |  | 7 | 5 OR 6 |
|  | Combining concepts | 8 | 4 AND 7 |
| Cochrane Library | HPV & Cervical cancer | 1 | Exp Human papillomaviruses/ |
|  |  | 2 | Exp Uterine cervical neoplasms/ |
|  |  | 3 | Papillomavirus Infections/ |
|  |  | 4 | HPV or human papillomavirus or papillomavirus infections or cervical cancer.tw,kf. |
|  |  | 5 | 1 OR 2 OR 3 OR 4 |
|  | Self testing | 6 | Exp self-testing/ |
|  |  | 7 | ((self or at-home or home or at home or self-administered or self-collect*) adj5 (test* or screen* or sampl* or swab* or collect*)).tw,kf. |
|  |  | 8 | 6 OR 7 |
|  | Combining concepts | 9 | 5 AND 8 |
| SocINDEX | HPV & Cervical cancer | 1 | HPV.mp. or exp Wart virus/ |
|  |  | 2 | cervical cancer.mp. or exp uterine cervix cancer/ |
|  |  | 3 | “Human papillomavirus".mp. |
|  |  | 4 | 1 OR 2 OR 3 |
|  | Self testing | 5 | exp self-testing/ |
|  |  | 6 | ((self or at-home or home or at home or self-administered or self-collect*) adj5 (test* or screen* or sampl* or swab* or collect*)).tw,kf. |
|  |  | 7 | 5 OR 6 |
|  | Combining concepts | 8 | 4 AND 7 |
